# Blockchain Applications in Health Care and Public Health: Increased Transparency

**DOI:** 10.2196/20713

**Published:** 2021-06-08

**Authors:** Pedro Elkind Velmovitsky, Frederico Moreira Bublitz, Laura Xavier Fadrique, Plinio Pelegrini Morita

**Affiliations:** 1 School of Public Health and Health Systems University of Waterloo Waterloo, ON Canada; 2 Center for Strategic Technologies in Health (NUTES) State University of Paraiba (UEPB) Campina Grande Brazil; 3 Institute of Health Policy, Management, and Evaluation University of Toronto Toronto, ON Canada; 4 Research Institute for Aging University of Waterloo Waterloo, ON Canada; 5 Department of Systems Design Engineering University of Waterloo Waterloo, ON Canada; 6 eHealth Innovation Techna Institute University Health Network Toronto, ON Canada

**Keywords:** health care, blockchain, EHR, health insurance, drug supply chain, genomics, consent, digital ledger, food supply chain

## Abstract

**Background:**

Although big data and smart technologies allow for the development of precision medicine and predictive models in health care, there are still several challenges that need to be addressed before the full potential of these data can be realized (eg, data sharing and interoperability issues, lack of massive genomic data sets, data ownership, and security and privacy of health data). Health companies are exploring the use of blockchain, a tamperproof and distributed digital ledger, to address some of these challenges.

**Objective:**

In this viewpoint, we aim to obtain an overview of blockchain solutions that aim to solve challenges in health care from an industry perspective, focusing on solutions developed by health and technology companies.

**Methods:**

We conducted a literature review following the protocol defined by Levac et al to analyze the findings in a systematic manner. In addition to traditional databases such as IEEE and PubMed, we included search and news outlets such as CoinDesk, CoinTelegraph, and Medium.

**Results:**

Health care companies are using blockchain to improve challenges in five key areas. For electronic health records, blockchain can help to mitigate interoperability and data sharing in the industry by creating an overarching mechanism to link disparate personal records and can stimulate data sharing by connecting owners and buyers directly. For the drug (and food) supply chain, blockchain can provide an auditable log of a product’s provenance and transportation (including information on the conditions in which the product was transported), increasing transparency and eliminating counterfeit products in the supply chain. For health insurance, blockchain can facilitate the claims management process and help users to calculate medical and pharmaceutical benefits. For genomics, by connecting data buyers and owners directly, blockchain can offer a secure and auditable way of sharing genomic data, increasing their availability. For consent management, as all participants in a blockchain network view an immutable version of the truth, blockchain can provide an immutable and timestamped log of consent, increasing transparency in the consent management process.

**Conclusions:**

Blockchain technology can improve several challenges faced by the health care industry. However, companies must evaluate how the features of blockchain can affect their systems (eg, the append-only nature of blockchain limits the deletion of data stored in the network, and distributed systems, although more secure, are less efficient). Although these trade-offs need to be considered when viewing blockchain solutions, the technology has the potential to optimize processes, minimize inefficiencies, and increase trust in all contexts covered in this viewpoint.

## Introduction

### Background

Global society is moving into an age of ubiquitous and smart technologies that monitor our health, such as smart devices, Internet of Things solutions, and ambient assisted living systems. These technologies allow continuous and effortless health data collection at a previously unseen scale [1,2], generating rich and massive data sets, known as big data [3].

The *age of big data* can lead to a change in the way health care is delivered. Generally, health care is reactive, in which individuals interact with health care services when there is something wrong [4,5] and usually to treat acute diseases, instead of proactive, in which real-time monitoring of health data from different sources leads to predictions and insights into individual and population health, as opposed to checkups with health services when a problem appears [4,5]. In this manner, a proactive and predictive health care model includes surveillance and monitoring of individuals through remote sensing technologies, such as smart bands and smart thermostats, generating large volumes of diverse and real-time data in a cost-effective manner. The use of such technologies in a community will also enable public health surveillance on a scale never seen before, allowing public health agencies to better understand the socioeconomic determinants of health and prevent disease outbreaks [5,6].

However, to achieve this model of health care, there are challenges that need to be overcome. For example, health records are stored by different providers in systems that lack interoperability [7,8]. This makes data sharing difficult and prevents doctors from having a complete view of a patient’s health [7,8]. Interoperability issues and costs also affect the availability of genomic data and minimize their benefits [9]. In addition, increasingly advanced methods of data collection and analysis of personal, medical, and genomic data raise concerns regarding ownership, privacy, and regulations of health data [1,3].

One possible tool to overcome or mitigate these challenges is blockchain [6,10-13]. This technology can be seen as a distributed virtual ledger that records timestamped transactions [6,12,13]. Cryptography is used to ensure that when a block is added to the blockchain, it cannot be tampered with [12]. Hence, blockchain is a tamperproof digital ledger in which all participants view an immutable version of the truth, making it ideal to track an asset and enable trust among parties (eg, health data or user consent for data collection) [6,7,12,14].

In 2016 and 2018, IBM Corporation surveyed more than 400 health care and life sciences executives on the use of blockchain technology. Among their findings, more than half of the executives in both industries had plans to adopt it by 2020 [6,10,11]. Given the perceived potential of blockchain by industry experts from multiple areas [6,10-12] and to help guide the implementation of digital solutions that can solve pressing needs in health care systems, the aim of this study is to review current blockchain solutions being developed by the health care industry. This paper provides a comprehensive view of the blockchain health care industry, providing guidance to innovators about how to leverage this technology in daily operations and how to implement solutions that can help evolve health care delivery. The COVID-19 outbreak has created an increased demand for home-based digital health solutions such as telehealth and telemonitoring [15], increasing the importance of using technologies such as blockchain to increase the transparency of digital transactions and data provenance [16,17].

### Related Work

McGhin et al [18] provide an overview of the main opportunities and challenges for blockchain in the health care field and describe some initiatives (both in academia and industry) focused on developing blockchain solutions. Vazirani et al [19] detailed a systematic review examining the feasibility of blockchain for electronic health record (EHR) systems, finding several trade-offs that need to be considered during the design and development of blockchain. Trade-offs were further explored by O’Donoghue et al [20].

Farouk et al [13] provided a similar review to this one on the use of blockchain in the health care industry but mostly focused on its integration with Internet of Things devices and record management. Hasselgren et al [21] conducted a scoping review of blockchain in health care and, while focusing on peer-reviewed publications rather than the industry, they found that both the number and quality of blockchain research is growing.

Chukwu and Garg [22] provide a systematic review of blockchain applications specifically for the use of EHRs and health data sharing and do not focus on industry applications.

Agbo et al [23] conducted a systematic review of blockchain applications in health care, also focusing on academic literature, although some studies mention companies working with blockchain. The use cases found in this work are very similar to the use cases explored in this paper (suggesting a convergence between academia and industry research), but Agbo et al [23] found a predominance of studies focusing on EHRs when compared with other areas.

Most of these did not have an industry focus; rather, they usually discussed the computer science aspects of the technology or evaluated mostly academic work. In addition, as found by Agbo et al [23], most reviews focused on EHRs and not on additional use cases. Therefore, this review contributes to preview work by providing an overview of blockchain applications in the health care industry, while identifying what challenges and use cases are the current focus of health care companies working with blockchain.

## Methods

### Overview

This narrative review [24] focuses on providing eHealth experts with a comprehensive narrative review of blockchain in health care. Blockchain is a novel technology that can provide increased transparency to data transactions in health care and public health [6,10,11]. Owing to its novelty and early stage implementation, significant development has been accomplished at the industry level, driving this review toward a combination of peer-reviewed academic literature and gray literature.

Our aim was to analyze blockchain in health care from an *industry* perspective, focusing on solutions developed by health and technology companies (although results from research and development initiatives and academia were used to complement knowledge when necessary).

Although not a scoping review, this paper followed the framework defined by Levac et al [25] for scoping reviews, ensuring that the findings were analyzed in a systematic manner. This framework consists of six stages: (1) identifying the research question (RQ); (2) identifying relevant studies; (3) selecting studies; (4) charting the data; (5) collating, summarizing, and reporting results; and (6) consultation (optional). In this narrative review, we leveraged phases 1-5.

### Identifying the RQ

The primary objective is to identify how the health care industry views the potential of blockchain to solve current challenges. To fulfill this, two secondary goals need to be achieved: we must understand how blockchain works and the challenges facing the industry. Therefore, the following RQs were used to guide the reviews:

1. How do the blockchain systems work?
2. What are the current challenges faced by the health care industry today that can be addressed by blockchain technology?
3. For each of these challenges, which blockchain solutions are being developed by the health care industry?

### Identifying Relevant Studies

Our review analyzes how the health care industry perceives the blockchain’s potential to solve current challenges. To this end, we looked at gray literature in addition to traditional databases such as IEEE and PubMed, including search and news outlets such as Google Scholar, CoinDesk [26], CoinTelegraph [27], and Medium [28]. The keywords were a combination of “blockchain,” “distributed ledger,” “health,” “industry,” and “health care.” Whenever possible, we looked at technical reports (usually available on companies’ websites) in addition to news articles.

### Study Selection

The primary exclusion criteria involved selecting solutions that address issues or challenges in health care. Blockchain solutions that only had applications in unrelated fields were not included. Additional restrictions included practical concerns regarding availability and language (only English references were included).

### Charting the Data

To extract useful insights from the publications, we focused on two main types of information:

- What are the main health care challenges that the solution aims to improve?
- How is blockchain being used to improve the challenges?

More specifically, we looked at the main objective of the blockchain solution and the methods in which blockchain is being developed. Relevant bibliographical information, including title, authors, country, and year, was also extracted. This review focused on technical reports. If the technical report did not provide sufficient information, web articles were used to complement the results.

### Collating, Summarizing, and Reporting the Results

Following the recommendations presented by Levac et al [25], the steps are as follows:

- Analysis: for each solution being presented, we mapped the challenges addressed and how blockchain is being used.
- Reporting results: after presenting additional information on blockchain, we will describe the challenge in question and its importance in health care, followed by a discussion on how blockchain is being used by the industry in this context.
- Implications for future research, practice, and policy: this final step will be addressed in the
  Discussion
  section, where we discuss the limitations of blockchain and additional concerns.

## Results

### Overview

We started this review by presenting relevant background information about blockchain, followed by an overview of the main challenges identified in our review: EHRs, supply chain, health insurance, genomics, and consent management. For each of these areas, we have also presented blockchain solutions developed by industry. Table 1 provides a summary of the results by describing each of the five identified challenges explaining how blockchain can offer a solution, along with examples.

**Table 1:** Results of the literature review.

Challenges	Description	Solutions
Electronic health records	Blockchain can provide an overarching framework that allows transparent and auditable access to disparate individuals’ health records stored off-chain. Patients would control data sharing parameters and access. Some solutions also discuss integrating health data from less traditional sources (eg, connected devices) and the creation of a health data marketplace, in which patients can sell their data to buyers through crypto tokens	MedRec [7,8,29], PatientTruth [30,31], CareX [32,33], MEDIS [34,35], GEM [36-40], MedicalChain [41,42], Humantiv and Medoplex [43-45]
Supply chain	Blockchain can establish an immutable record of a product’s tracing throughout the supply chain. In the case of health care, there have been many solutions that implement a blockchain to track-and-trace drugs and food products. In addition, smart contracts can be used as monitoring and alert systems for proper transport conditions (eg, a certain temperature range)	Drug supply chain: BlockVerify [46-48], Merck [49,50], Modum [51-54] Food supply chain: IBM Food Trust [55-58], Alibaba and Ant Financial [59,60]
Health insurance	Smart contracts on the blockchain can potentially help to settle health insurance claims and manage payment in real time, making the process more efficient and transparent for payers, providers, and patients. Other potential use cases include pharmaceutical and medical benefits, checks, and payment risk calculation	PokitDok and DokChain [61-67], GEM [39], Payspan [68,69]
Genomics	Much like with electronic health records, blockchain can provide a mechanism for controlling access to separate existing data banks of genetic information. In addition, blockchain can directly connect sellers of genomic data-to-data buyers, creating a genomic data marketplace. Data buyers could even provide rewards for individuals to sequence their genomes, creating their own data sets (eg, providing crypto tokens to individuals with a certain feature to be researched, in return for their genomic information)	Nebula Genomics [9,70], LunaDNA [71-75], Shivom [76-79], Zenome [80,81], EncrypGen [82-85], Macrogen [86-88]
Consent management	Blockchain can provide an immutable and timestamped log of consent, allowing individuals to grant and revoke consent for different data types and periods. In the case of health studies, it can also help researchers to easily track, manage, and update user consent	My31 app [89,90], Bitfury [91,92], HealthVerity Consent [93], Verifiable Audit Trail (tracking of events related to health data) [94-98], INSERM^a^ and APHP^b^ consent project [14], Queen’s University BlockTrial [99], Patient Control and Consent Blockchain initiative [100-102], Ubiquitous Health Technology Lab [6,103]

^a^INSERM: Institut National de la Santé Et de la Recherche Médicale.

^b^APHP: Assistance Publique-Hôpitaux de Paris.

### What Is Blockchain?

Blockchain is a virtual distributed ledger that records transactions among parties. It is operated by a network of computers in which each participant is called a node and possesses a copy of the ledger, regularly updated to ensure consistency. In other words, all nodes have access to the exact information [12,18].

When a user makes a transaction, this transaction is timestamped and *sealed* in a block [12]. Through a consensus mechanism, this block is linked to previous existing blocks—hence the name blockchain. Different blockchains (eg, Bitcoin and Ethereum) have different consensus mechanisms [12]. A typical consensus mechanism, called Proof of Work, requires the nodes in the network to randomly guess a number that solves a mathematical puzzle; the first node to discover it seals the block. This process is called mining [6,12,13,18].

**Figure 1:**
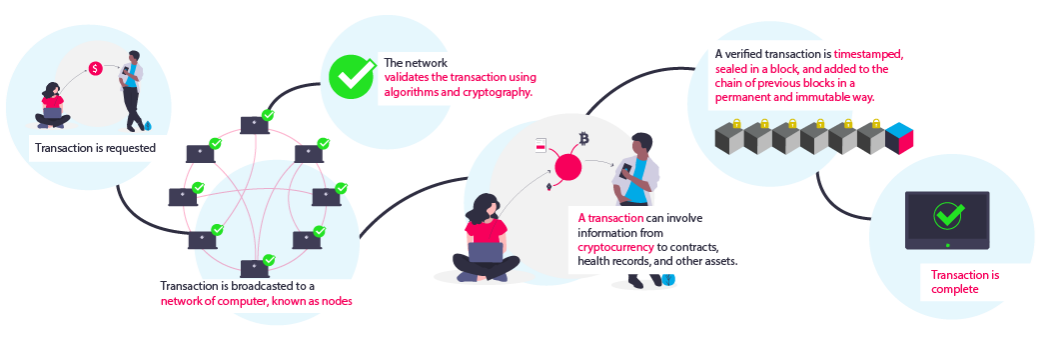
Flow of a transaction in the blockchain.

The linkage between blocks is achieved through a method called hashing, in which new blocks point to the previous ones [12]. This technique converts data into a string of characters, called a hash. For example, a user may convert a text into the following hash: “f1abc234b79f6d6ay42a12c53468a1b13553r1r0fgr4039 rf08h958b5232b9n8.” If a single character from this hash is changed, an entirely new string is generated. Although it is easy to generate a hash from a piece of information, it is impossible to discover the original information from a hash [12,22]. The Bitcoin blockchain hashes the nonce, alongside the transaction information and the hash of the preceding block. If a malicious party tries to tamper with information already stored in a block, the hash is altered, breaking the chain. This ensures that the blocks cannot be tampered with, and the information contained in the blockchain cannot be altered. Therefore, blockchain is a tamperproof digital ledger where all participants have access to an immutable version of the truth [12,18]. The flow of a transaction in the blockchain is shown in Figure 1.

Blockchain is a type of distributed ledger technology, in which a consistent ledger is shared among parties to store a record, creating a distributed database. It is a distributed ledger technology that uses cryptographic and consensus mechanisms to increase trust [22].

There are also different types of blockchains. Although the nomenclature varies, they are usually defined as follows [13,104,105]:

- Public blockchain: all participants can read and write new information to the ledger. Although new information can be added, no information can be deleted. Bitcoin is an example of a public blockchain.
- Permission (consortium and federated) blockchain: this is owned by a consortium of participants who define the permissions for joining and updating the network. For example, a consortium blockchain owned by health care providers can allow patients to change their information, but only providers may upload new information.
- Private blockchain: this is owned by a single entity that manages access, permission to read or write data, and even data deletion. Among the blockchain communities, some are of the opinion that private blockchains defeat the purpose of decentralized technologies by introducing a central authority.

From a health care perspective, one of the biggest concerns in capturing and coding patient information is privacy [106]. Several blockchain implementations allow the creation of smart, codified contracts that allow for the storage of immutable information. For example, Ethereum enabled the creation of smart contracts that codify contract agreements. When several parties agree to a transaction, they create mechanisms to ensure trust [6,12,107]. Smart contracts write the terms of a contract in code, which is executed on the blockchain, and has *the ability to be self-executing and self-enforcing* [12]. Therefore, smart contracts can minimize trust concerns among parties [12,18,107].

Blockchain’s features and design make it a model for processes plagued by trust issues [6,12,108], and it is ideal for increasing trust in contexts involving parties that do not have reason to trust each other [6,12,108]. One such context is health care [6].

### Blockchain in Health Care: Challenges and Solutions

The following subsections describe a challenge in health care and discuss blockchain solutions being developed by companies to address them.

#### Electronic Health Records

##### Challenge Description

EHRs digitally store patients’ health data [6,7,109,110]. However, data are fragmented throughout EHR systems: patients often interact with different health care providers (usually the stewards of the data), creating challenges related to accessing past information [6,7,22]. In addition, providers have different EHR systems that may not be fully interoperable [6,7]. These factors contribute to difficulties in data sharing [6,7].

Patients’ health data end up in silos and cannot be integrated with data from other providers or sources, such as connected devices. Ultimately, there is no easy way to obtain a holistic view of a patient’s health, leading to errors, delays, and poorer health outcomes [6,7,18,110].

##### Use of Blockchain

Blockchain solutions can create an overarching hub, potentially on the cloud, to link all records of individual patients [7,8,18,29], without storing health data on the blockchain itself [7,8,29,111]. Rather, the blockchain infrastructure would act as a hub that points to the location of a patient’s records off-chain [7,8,29]. Data access and changes to records can be tracked and displayed to the patient in real time. Furthermore, patients could control access to their records by giving permission to providers, researchers, and third parties to access their data. In this manner, an EHR-blockchain solution would allow for all health data from individuals to be accessed and controlled by the patient, facilitating a complete view of patients’ health [7,8,18,29]. This solution would also give patients greater control and transparency over their health data [7,8,12,18,19,29].

For example, MedRec is a blockchain-enabled solution for EHRs [7,8,29]. It is a system developed by the Massachusetts Institute of Technology that provides a transparent view of medical history. MedRec uses smart contracts in Ethereum to encode metadata by referencing medical data from different sources, including information about ownership and permission. These references “create an accessible bread crumb trail for medical histor[ies]” [7]. Providers may append a new patient record in MedRec, but patients are the ones who give permission for data to be accessed and shared. This increases transparency and allows patients to keep track of their records [7,8].

Similar solutions include PatientTruth [30,31], CareX [32,33], MEDIS [34,35], and MedicalChain [41,42]. Typically, EHR-blockchain solutions store references to off-chain files containing EHRs and also work with less traditional sources, such as data from connected devices. Patients control their records and with whom they wish to share their data (eg, health care professionals, hospitals, and insurance providers). Some of these solutions allow patients to sell deidentified records (eg, to health studies) with a crypto token from the platform. The financial component creates a form of health data marketplace in which patients own and are able to profit from their health data.

Another organization working with blockchain in an EHR context is CitizenHealth [33,43], which developed two solutions: Humantiv [44], which also combines data from EHRs and other sources with an added gamification component in which patients earn rewards according to their health indicators, and Medoplex [45], the company’s marketplace component. GEM, a US-based start-up, is developing a solution that uses Ethereum to create a shared network where providers have real-time access to medical documents [18]. GEM is partnering with Nordic-based Tieto to create a blockchain platform that enables patient control over medical records and genomic data [40,112,113].

It is important to note that a blockchain infrastructure, as described above, could mitigate data sharing issues by providing an interoperable, auditable, and secure landscape of transactions controlled by data owners. This, in turn, would allow easy and transparent access to disparate health records. As stated by McGhin et al [18], when discussing blockchain cloud infrastructures, “the role of blockchain in cloud data infrastructure is facilitating the creation of a decentralized and trusted cloud data provenance architecture that allows tamperproof records, greater transparency of data accountability, and enhanced privacy and availability of the data.” However, the blockchain itself does not impact the interoperability of the health data itself or the local systems in which it is stored. Rather, it acts as an overarching infrastructure with references to off-chain resources whose access is auditable, secure, and transparent to all authorized parties within the distributed network.

#### Drug Supply Chain

##### Challenge Description

One of the biggest challenges faced by pharmaceutical companies today is counterfeit drugs. In total, US $200 billion are lost to counterfeit drugs annually, and their use puts patients’ lives at risk [46]. Manufacturers do not have a unified and interoperable system of supply chain management, lack incentives to share data and information, and are consequently siloed, making end-to-end traceability and drug provenance difficult [46,114].

In the United States, the Drug Supply Chain Security Act (DSCSA) established a set of requirements that must be implemented by pharmaceutical companies until 2023. These requirements include product tracing and verification [46,115,116].

##### Use of Blockchain

By storing transactional data from the supply chain on blockchain, it is possible to establish an *immutable record of provenance* [117]. Blockchain can provide a transparent ledger that traces products throughout the supply chain, from manufacturing to distribution. This will ensure compliance with the DSCSA and improve patient safety. Furthermore, blockchain can also track whether products are being transported and handled under appropriate conditions [111].

One of the companies working in this scenario was BlockVerify. The company is working on a DSCSA-compliant solution that traces products and identifies counterfeit drugs [46-48] A product is labeled with BlockVerify’s tag and verified along the supply chain, with a permanent record on a private blockchain. Consumers and retail locations can use this record to ensure that the product is genuine. Merck has filed a patent to use blockchain to track drug information in the supply chain; while the company already has systems to prevent fraud, it is expected that blockchain could minimize existing inefficiencies [49,50]. Modum uses blockchain to ensure that medical products are being transported at the correct temperature [51-54]. Sensor devices are added to shipments, and smart contracts for each shipment are fixed with sensors, including the alarms. The sensors monitor the temperature during transportation and, when the shipment is received, the data are transferred to a blockchain and the smart contract evaluates whether conditions and regulations have been met [51-54].

Efforts have also been made to tackle the food supply chain. IBM Food Trust is a permissioned blockchain that allows stakeholders to view the supply chain history of a food item and complementary information (eg, certifications, testing, temperature, and location). For example, organizations can identify when a food item is contaminated and trace the contamination back to its source [55-58]. Another solution is being used in China: Ant Financial, an affiliate of the e-commerce enterprise Alibaba, is using a permissioned blockchain that tracks the production of rice in the city of Wuchang. Quick Response codes were added to rice packages, so that users scan these codes to obtain information (eg, location of the harvest, type of seed used, or transportation). Alibaba is also working on developing a food trust framework that, in its pilot phase, tracked shipments from China to Australia and New Zealand [59,60].

#### Health Insurance

##### Challenge Description

There is a lack of trust between payers, providers, and patients with a complete view and coordination of health care [6]. Patients often pay expensive premiums while dealing with a lack of transparency and the ability to compare prices, in addition to the risk of insurance fraud that affects all stakeholders [32,41,69]. Providers must go through complex and bureaucratic processes to submit a claim [41]. The challenging ecosystem can be exemplified through the claims management process: (1) first, a provider must be covered by health insurers—meaning that the provider must also maintain benefits’ databases and keep track of services delivered, adding additional expenses; (2) if patients receive services from the provider, the insurer checks the service against the patients’ health plans to check their eligibility; (3) the process takes several weeks and involves multiple people checking agreements, leading to delays [41].

##### Use of Blockchain

In 2017, a survey revealed that 98% of payers with over 500,000 members are pursuing blockchain-enabled solutions [69]. Through the use of smart contracts, it is possible to codify terms of agreement between providers, payers, and patients, automating processes and minimizing inefficiencies. For example, PokitDok developed DokChain, a private blockchain that references off-chain file systems. DokChain contains several smart contracts that request and return data from health insurance providers and payers in real time [61-63], possibly enabling real-time status checks and mechanisms for error identification. The goal is for DokChain to make decisions on insurance claims in real time using smart contracts. As soon as a patient receives a service, it is recorded on DokChain and visualized by all stakeholders. Smart contracts can determine whose responsibility the claim falls under and how much is owed to each party, processing that amount in real time [62,64,65]. PokitDok also offers application programming interfaces to calculate payment risk for a patient [66] and to allow patients to schedule and pay for health services [67], automating payments and checking benefits in real time.

The start-up GEM created a blockchain prototype in 2017 that helped to settle a claim in less than 5 minutes [39]. Payspan, a 25-year-old health care reimbursement company, is also working on blockchain networks to connect providers and payers for claims management and payment processing [68,69].

Initiatives that focus on the creation of a health data marketplace (eg, Medoplex) want to allow health care buyers (eg, patients) and sellers (eg, providers) to connect without the need for insurance companies. Their goal is to create a marketplace platform where patients can search, select, and pay for health care services using a crypto token. CareX, MedicalChain, and BlockRx are other solutions that allow for the payment of health services with crypto tokens obtained from health record sharing [32,41,46,111,114]. Many companies that deal with EHRs seem to view health insurance as a complementary use case: by creating a health data marketplace platform that allows patients to share their data with health care providers and professionals, the idea seems to be that intermediaries (eg, insurance companies) will not be needed or their role will be diminished.

#### Genomics

##### Challenge Description

DNA is a molecule that encodes the genetic instructions of organisms [9], where genes are collections of DNA [118] and a genome is the collection of an organism’s genes [9,119]. The human genome comprises more than 20,000 genes [9,81,120,121].

The study of genomics data in conjunction with social and environmental determinants of health gave rise to precision medicine [122]. This field of research and treatment can help individuals and researchers better understand the cause of diseases, contribute to the development of new drugs, and aid in the creation of personalized interventions for individuals with specific genetic traits, in addition to many other benefits [123]. However, for this potential to be fully realized, large volumes of genomic data are required [9,124].

There are several barriers to the availability of massive genomic data sets, including security and privacy concerns, prohibitive costs, and data sharing [9]. The latter is related to a lack of interoperability between systems that store genomic data and to data ownership [9]. In addition, a human genome generates over 200 GB of data, and it is estimated that more than 100 million genomes will be sequenced by 2025. Therefore, storage and network transfer speeds also limit data sharing [9,81,121].

##### Use of Blockchain

The business model for genomic data involves individuals hiring companies such as 23andMe and Ancestry to sequence their genome and receive results. These companies then sell the sequenced data to researchers. With a blockchain network that connects data sellers (eg, an individual who sequenced their genome) and data buyers (eg, a pharmaceutical or research company), without the need for personal genomic companies acting as intermediaries, the transparency of the process will increase while costs will decrease. In addition, data sharing problems can be improved by connecting disparate genomic records on the blockchain, similar to what MedRec and other blockchain-EHR solutions have been proposing [9,111]. Although this will not solve all interoperability issues in the industry, it will allow easier, secure, and transparent access to disparate records of data, facilitating data sharing.

One of the companies that hope to build a genomic and health data marketplace is Nebula Genomics, through the development of a *storage, sharing, and computing platform for biomedical big data* [70]. The company has a partnership with Veritas Genomics. Veritas’ platform processes and stores large amounts of genetic information, and Nebula hopes to build on top of it. Individuals will be able to store their genetic information on Veritas’ platform and, with blockchain, share and sell their data in the genomic data marketplace. Similar to the case with EHRs, users own their data and control permissions for data sharing, increasing transparency and minimizing security and privacy concerns. Their proposed solution has the potential to [9] (1) minimize costs, since data buyers will acquire data directly from owners who can receive sequencing subsidies from buyers to encourage the generation of genomic data sets (eg, offering sequencing subsidies to individuals with specific traits that a specific research group plans to study); (2) increase transparency, since owners will control access and data sharing will be protected through cryptography. Owners will remain anonymous, while buyers will have to provide information about their identity. Blockchain records all transactions in an immutable log that is easily auditable; and (3) increase the availability of data by integrating data from several sources. The network offers space-efficient data-encoding formats and leverages computer resources to facilitate the transfer of information [9,70,121].

Other companies have similar solutions, with minor differences. LunaDNA allows anyone to join the blockchain network and win company shares based on shared data in the form of a crypto token. Researchers pay to conduct research on aggregated data, and proceeds earned from research are passed on to stakeholders as dividends. Unlike Nebula Genomics, LunaDNA currently does not provide genome sequencing services, but it accepts files from companies such as 23andMe and Ancestry. Blockchain is used to give individuals ownership of their data and for the generation of an immutable log of transactions [71-75]. Shivom [76-79], Zenome [80,81], EncrypGen [82-85], and Macrogen [86-88] are other similar companies that are using blockchain to empower patients by allowing them to own and share genomic data.

#### Consent Management

##### Challenge Description

In health studies, the process of obtaining consent from participants is fallible [125]. The Food and Drug Administration cited the main deficiencies related to consent: the failure to obtain informed consent, use of expired or incomplete forms, failure to provide copies of the forms to participants, missing documents, and changes made to documents without the approval of a review ethics board [14,125]. These problems are aggravated given that reconsent has to be sought in several cases (eg, when there is a revision of the study protocol, new risks are discovered, or there is a worsening of the medical condition of a participant) [126]. Oftentimes, consent needs to be obtained for cohabitants, caregivers, or legal guardians [125-128].

In addition to limitations in traditional consent methods, the global society is moving into an age of ubiquitous smart technologies that monitor our health, which increases the complexity of data collection points and, in turn, of consent management. The challenge of obtaining consent for increasingly advanced methods of data collection, use, and disclosure calls for new solutions to perfect consent procedures and the need to protect the safety of individuals.

##### Use of Blockchain

Blockchain can provide an immutable and timestamped log of consent, making the process more transparent [6,12,14,111,129]. In the case of health studies, participants will be able to monitor and manage consent, giving informed consent for certain types of data to be collected but not others and revoking their consent at any time. Through the use of cryptographic techniques of identity management, participants can make sure that they are reviewing the latest consent forms and that these were approved by the review ethics board. Researchers would also be benefited, as the measures taken to ensure ethical and legal requirements throughout the research would be clearly auditable. Moreover, it would be easier for them to obtain, track, and update patient consent during the study.

With a consent management platform built on blockchain, authorized parties in the network will have access to timestamped and tamperproof logs of user consent. However, this does not serve as a magic bullet to solve all consent management issues, and researchers must still be careful when collecting informed consent and ensure all ethical and legal requirements. For example, participants may revoke their consent at any time during the study (revocability), and researchers would need to stop collecting participant data at this point despite the fact that data may already have been collected (nonretractability). Similar to traditional health studies, consent forms should include a description of the protocols necessary for revoking consent according to data protection regulations and indicate to participants whether their data are being deleted after their consent is nullified. For example, Article 17 of the General Data Protection Regulation describes the “right to be forgotten” [130], in which personal data must be deleted after consent is revoked.

This would not present a problem for blockchain systems, as the collected data would not be stored on the blockchain, rather the consent of the individual is. If a participant revokes consent, the system will instantly be updated to reflect this and notify the researchers of the change. All consent status updates of participants cannot be tampered with on the blockchain and can be easily auditable in case of problems [129]. Furthermore, if the study uses connected devices for data collection, smart contracts can be developed to ensure that, as soon as a consent is revoked, data immediately stop being collected. What will happen to the data that were already collected depends on the study protocol designed by the researcher. If data deletion is needed, the researcher will have to delete the data manually from their own database (which is not related to blockchain in any way; as mentioned, blockchain only stores the information on consent and not the data as such). In this way, a blockchain-consent platform can facilitate the process of consent management and mitigate some trust issues, but it is still up to researchers to ensure correct and ethical protocols and guidelines are being followed.

Hu-manity.co developed a mobile app called My31, built on IBM blockchain, to help individuals manage their consent to the use of their personal and health information. Users can consent to sharing their data with third parties and researchers, and receive compensation for it [89,90]. Another blockchain-powered platform for managing consent is being developed by Bitfury, with the goal of managing consent for research. All updates related to user consent are timestamped and recorded on the blockchain for future auditing [91,92]. The solution can be used both as a new system and in conjunction with existing systems. HealthVerity is also developing a blockchain solution to manage consent. Unlike other solutions, HealthVerity’s consent platform seems to be more focused on consumer applications that collect health data than medical or clinical research [93].

Although not specifically on consent management, DeepMind (Google’s artificial intelligence conglomerate) is developing a project to allow hospitals and patients to track events related to health data in real time [94-98]. Any interaction with a patient’s health data is recorded on a distributed ledger, which will store information stating that the data were used and their purpose. This project, *Verifiable Audit Trail*, has the potential to increase transparency in health research and minimize some of the trust issues between stakeholders [94-98].

There have also been several academic initiatives exploring the use of blockchain for consent management. For example, researchers from Institut National de la Santé Et de la Recherche Médicale and Assistance Publique-Hôpitaux de Paris in France have created a proof of concept to manage patient consent in clinical trials that use cryptographic signatures for e-signing. The timestamped consent for different form versions is recorded on the blockchain as a master document [14]. In Canada, Queen’s University has also developed a similar solution for clinical trials, titled BlockTrial, where patients assign permission for data access and researchers can query off-chain data [99]. The Toronto-based University Health Network, in partnership with IBM and digital health agencies, is working on the patient control and consent blockchain initiative to allow the permissioned access of data, managed by patients through individual consent and built on blockchain to allow immutable storage of consent directives. Patients can access a mobile app, managing who has access to their data and for what reason. Currently, only University Health Network–produced data are available, but the goal is to enable the integration of data from multiple sources [100-102].

Velmovitsky et al [129] also developed a proof-of-concept blockchain to help patients manage consent, focusing on third-party consumer apps that collect health data such as smart devices. In this prototype, patients can grant and revoke consent for different data types and different periods (eg, users give consent for temperature to be collected but not movement, from September to October). This work uses Hyperledger Fabric to create a blockchain network and proposes a governance structure to collect data from smart devices.

## Discussion

### Blockchain in Health Care

The health care challenges in the areas of EHRs, supply chains, health insurance, genomics, and consent management are not mutually exclusive. For example, the use of blockchain to address challenges in genomics and EHRs shares many similar challenges regarding data ownership, data sharing, and the creation of a marketplace where owners and buyers can trade data. Consent management and EHRs can also be complementary, with blockchain-enabled solutions for consent acting as a sort of *access control*. Researchers and health care providers could potentially access individual health records stored in off-chain databases through blockchain infrastructure, provided that they have consent from the individual.

It is interesting to note that many of the solutions described seem to consider only an ideal workflow. In an emergency situation where the patient is unable to authorize data sharing, there should be mechanisms for doctors and nurses to access data [18].

Similar to any new technology, while blockchain seems to hold the potential to improve several existing challenges in health care and give more agency to patients, the actual impact of the technology is unclear. Many solutions want to eliminate intermediaries (eg, allowing patients and providers to connect without the need of insurance companies; however, it is impossible to determine whether this kind of disruption will actually happen and whether patients are ready for such complexity [6,12,18]). Although blockchain solutions can minimize inefficiencies, they are more likely to run in conjunction with existing systems from third parties. In other words, the solutions will incorporate third parties and make the process more transparent for the participants, thereby increasing trust [12]. This would also ease the adoption of blockchain in the industry, as several stakeholders would offer less resistance.

Most of the solutions we found, with a few exceptions, seem to be at the initial development or prototyping stage: their architecture and basic functionalities are planned out, and companies are now looking to raise funds and continue their implementation. Although this shows that the health care sector is positive about the potential of blockchain, it also makes it difficult to concretely evaluate the potential of the technology.

### Immutability, Decentralization, and Trust

Blockchain is designed to be distributed, transparent, and immutable by design [6,12] and can improve trust among stakeholders [6,12,18,108]. This is true in the scenarios described above. By eliminating centralized decision making, automating processes, and increasing transparency in data collection and use, blockchain can increase trust in health care processes.

However, blockchain’s embedded features could prove to be a challenge if misused. For example, the fact that every node in the network maintains a copy of the ledger is redundant. Decentralized systems are less efficient, scalable, and cost-effective compared with centralized systems [12]. By design, every participant node must possess a full copy of the distributed ledger; as the number of participants increases, so does the computational requirements such as storage and energy. Blockchain has been seen as a potential solution to address issues in the environment, such as creating a marketplace for energy trading. However, some critics point out that blockchain will do more harm than good because it expends a huge amount of energy [6,99]. These factors may limit the scalability of the blockchain solutions. They are not limitations of the technology per se, but design characteristics, and developers must consider a trade-off between these and the desired transparency and security provided by the distributed ledger. Trade-offs between different blockchain features must also be considered (eg, using a public or permissioned blockchain), as explored by O’Donoghue et al [20].

Similarly, blockchain’s immutability means it is *append-only* for public implementation [12]. Companies dealing with General Data Protection Regulation must abide by a key principle of regulation, which is the right to data deletion [12,19,131]. However, this is not an option for public blockchain implementations [12]. This is one of the reasons why it is not recommended to store personal data on the blockchain [7,19]. Rather, it should be stored in off-chain databases with reference to the chain. A study conducted by Park et al [132] studied the feasibility of storing, sharing, and managing records on the blockchain. The study confirmed that it was possible to manage records in a private blockchain, but several challenges need to be addressed first, such as data size and costs.

Although blockchain provides an immutable ledger, it should also be noted that the information stored in the ledger is only as accurate as its input. This means that developers should create mechanisms to ensure that the correct data are uploaded to the blockchain [18,99].

### Key and Identity Management

Information stored on the blockchain is secured through cryptographic techniques that require the use of public and private keys [18]. Private keys act as a password that allows the user to access information. For example, in the Bitcoin blockchain, user A can send Bitcoins to user B using B’s public key. However, user B will only be able to access the Bitcoin with their private key. Users have one key to access all their blocks (analogous to a password to access all user data). If the key is compromised, all user data may be leaked. In the case of health care, which deals with sensitive data, this issue should not be understated [18]. For blockchain-EHR solutions, if a malicious party obtains a patient’s private key, there is a significant risk of identity theft, as they will have access to the patient’s full medical history [18].

One possible alternative to key management is through identity verification using blockchain [18,62,131]; it can provide an immutable ledger that stores and maintains legal documents such as birth certificates and business contracts. A person would then be able to prove their identity by accessing the blockchain [18]. Many countries, such as Estonia, use blockchain to verify various attributes of its citizens [12,131]. By being able to say if a person is an Estonian resident through blockchain, the platform can provide additional web-based services (eg, allowing citizens to vote on the web). The country already has an EHR system, accessed by a unique ID that uses blockchain to ensure integrity [10,131]. PokitDok is studying the application of a protocol called the contextually relevant identity management protocol with DokChain, in which different aspects of an identity are used for validation. For example, to buy an alcoholic a drink, the only necessary information is age. DokChain’s solution would allow users to decide which specific personal information to share based on the context. To validate an identity, the technology is planning to create a consensus mechanism in which an individual’s private key is partitioned among third parties. The key can be regenerated by using a subset of partitions. This is initially used for key recovery, but the goal is to have this function as implicit identity verification every time an identity needs to be validated [62]. A similar solution that distributes a user’s identity attributes is being developed by the Canadian company SecureKey [131].

### Conclusions

Blockchain, an immutable ledger in which all participants view an immutable version of the truth, is a promising technology that can help to minimize several challenges currently experienced in health care. This paper focused on a literature review and market assessment to determine the main challenges that could be improved by blockchain in today’s health care industry. The results showed the following five challenges:

- Health records are stored between different providers in systems that lack interoperability. This makes it difficult for stakeholders to share data and, therefore, to have a complete view of a patient’s health.
- Provenance and counterfeit products in drug supply chains are among the biggest hurdles faced by pharmaceutical companies today.
- There is a lack of connectivity among payers, providers, and patients in terms of insurance, which prevents access and coordination of care.
- Genomics data, one of the greatest promises of precision health, are limited by high costs, lack of massive data sets, and interoperability issues.
- Increasingly advanced methods of data collection and analysis raise concerns regarding ethics, ownership, privacy, and regulations of health data.

Although solutions vary, blockchain can provide an immutable and tamperproof log of transactions. Blockchain’s features of immutability, decentralization, distribution, and transparency can optimize processes, minimize inefficiencies, and increase trust in all contexts covered in this review. However, no silver bullet to solve every need in health care exists, and companies, developers, and decision makers need to be careful when considering a blockchain solution. Although the technology has potential, it also brings new concerns about data ownership and security.

### Limitations

As the aim of this review was to provide an industry point of view of the use of blockchain in health care, when defining the protocols for inclusion and exclusion, we did not include a critical component to evaluate the solutions included. Furthermore, while we searched for white papers and reports of the solutions whenever possible, we included in our results solutions already in place, in early stages of development, or recently announced, as our goal was to provide a mindset of the health care industry pertaining to blockchain. Future work should focus on critically analyzing the solutions described here for feasibility and efficiency. An interesting consideration from this analysis would be to define which challenges can be further improved with the use of blockchain. In other words, this analysis could point to where blockchain would be the most useful. In addition, blockchain can be used in conjunction with new fields of computer science, including big data and artificial intelligence [6,117]. Future work should also focus on studying blockchain in the context of these emerging technologies. Finally, it is important to note that the ontology used here, dividing the topics into five major challenges, is not exhaustive. There are many use cases where blockchain can be applied that, while not directly related to health care, may affect it (eg, key and identity management). In addition, many challenges described in the literature (eg, interoperability and data sharing of health systems) are encompassed by this ontology. In exploring these five areas, our aim was to provide an overview of the solutions being developed by the health care industry and which areas they are directing their efforts, rather than providing a defining list of use cases in health care in which blockchain can be used.

## Abbreviations

DSCSA: Drug Supply Chain Security Act
EHR: electronic health record
RQ: research question
